# A Protein Allergen Microarray Detects Specific IgE to Pollen Surface, Cytoplasmic, and Commercial Allergen Extracts

**DOI:** 10.1371/journal.pone.0010174

**Published:** 2010-04-16

**Authors:** Katinka A. Vigh-Conrad, Donald F. Conrad, Daphne Preuss

**Affiliations:** 1 Department of Human Genetics, The University of Chicago, Chicago, Illinois, United States of America; 2 Department of Molecular Genetics and Cell Biology, The University of Chicago, Chicago, Illinois, United States of America; Cairo University, Egypt

## Abstract

**Background:**

Current diagnostics for allergies, such as skin prick and radioallergosorbent tests, do not allow for inexpensive, high-throughput screening of patients. Additionally, extracts used in these methods are made from washed pollen that lacks pollen surface materials that may contain allergens.

**Methodology/Principal Findings:**

We sought to develop a high-throughput assay to rapidly measure allergen-specific IgE in sera and to explore the relative allergenicity of different pollen fractions (i.e. surface, cytoplasmic, commercial extracts). To do this, we generated a protein microarray containing surface, cytoplasmic, and commercial extracts from 22 pollen species, commercial extracts from nine non-pollen allergens, and five recombinant allergenic proteins. Pollen surface and cytoplasmic fractions were prepared by extraction into organic solvents and aqueous buffers, respectively. Arrays were incubated with <25 uL of serum from 176 individuals and bound IgE was detected by indirect immunofluorescence, providing a high-throughput measurement of IgE. We demonstrated that the allergen microarray is a reproducible method to measure allergen-specific IgE in small amounts of sera. Using this tool, we demonstrated that specific IgE clusters according to the phylogeny of the allergen source. We also showed that the pollen surface, which has been largely overlooked in the past, contained potent allergens. Although, as a class, cytoplasmic fractions obtained by our pulverization/precipitation method were comparable to commercial extracts, many individual allergens showed significant differences.

**Conclusions/Significance:**

These results support the hypothesis that protein microarray technology is a useful tool for both research and in the clinic. It could provide a more efficient and less painful alternative to traditionally used skin prick tests, making it economically feasible to compare allergen sensitivity of different populations, monitor individual responses over time, and facilitate genetic studies on pollen allergy.

## Introduction

Allergy affects 10–40% of the population [1] and results in elevated IgE [2] - a condition that is often diagnosed with skin prick tests (SPT) that can cause discomfort, risk anaphylaxis [3] and can increase patient sensitivity to allergens [4]. Safer and more quantitative alternatives for measuring allergen-specific IgE include enzyme linked immunosorbent assay (ELISA) and radioallergosorbent test (RAST) [5], yet their prohibitive cost limits use to select allergens and to severely affected patients. Microarrays potentially provide a more affordable diagnostic, and recent studies, most of which focus on recombinant allergens, have demonstrated their analytical and clinical feasibility [4], [6]–[16].

Considerable effort has been invested in identifying allergens, with the goal of providing more accurate patient diagnosis and the development of safe and effective immunotherapy treatments [17]. According to the International Union of Immunological Societies Allergen Nomenclature Sub-Committee, over 600 allergens have been identified to date (http://www.allergen.org). Most of these were identified by immunoblotting soluble allergen extracts separated by electrophoresis with patient sera or monoclonal antibodies [18]. Allergens identified in these studies are typically 10–70 kD cytoplasmic proteins and have diverse biological functions [2], [18], [19]. Despite current successes, it remains important to continue the search for new allergens, especially from the pollen surface, which has been largely overlooked in the past.

The pollen extracellular matrix plays an essential role in plant reproduction [20] and numerous studies have focused on purifying and characterizing many pollen surface materials [21]. While some researchers have suggested these materials also play a role in allergy [22]–[25], in most cases, the allergenic role of the pollen surface has not been explored. This is perhaps because commercial suppliers of pollen extracts used for research (e.g. identification of allergens), as well as diagnostics (e.g. SPT and RAST) and treatment (e.g. immunotherapy), wash pollen with organic solvents such as ether and acetone before extraction to remove possible contaminants, including microbes [26]. This strips away molecules from the pollen surface - a multilayered structure comprised of an internal cellulose layer (intine), an outer (exine) wall, and an extracellular matrix (the pollen coat) containing lipophilic proteins, lipids, and small molecules [27], [28]. Pollen coat components are primarily synthesized by anther cells that surround developing pollen grains [29], not the pollen itself [30]. Consequently, whether pollen allergens are identified by immunoblotting soluble protein extracts from commercially washed pollen [31], [32] or by screening expression libraries derived from pollen cDNAs with patient sera [33], pollen surface materials are likely to be overlooked. This is likely to account for the observation that many previously identified pollen allergens can be localized to the pollen cytoplasm [2], [19].

Here, we separately examined extracts of the pollen surface and cytoplasm from a wide range of plant species, taking care to collect material from unwashed pollen grains and to solubilize lipophilic pollen fractions. These extracts were arrayed along with commercially available allergens in microarray format that can detect allergen-specific IgE using only small amounts (<25 uL) of sera. We screened sera from 176 individuals with elevated total IgE and demonstrated that the allergen microarray is a reproducible measure of allergen-specific IgE. This assay provides extensive allergen sensitization information while using only limited resources, supporting the hypothesis that allergen microarray technology is a more efficient and economically feasible diagnostic that currently used approaches.

## Materials and Methods

### Ethics Statement

This research was approved by the University of Chicago IRB and documentation of this approval is available from their office under protocol #15100B. Sera from patients were de-identified and data was analyzed anonymously.

### MIAME compliance and data availability

The microarray experiments described in this manuscript are MIAME compliant and the raw data has been deposited in ArrayExpress (http://www.ebi.ac.uk/microarray-as/ae/); accession number E-MTAB-177.

### Allergen extract preparation

Raw un-defatted pollen from 22 allergenic plant species, allergen extracts of the same pollens, and allergen extracts of non-pollen allergens were purchased from Greer (Lenoir, NC). For a complete list, including full names, scientific names, and abbreviations, see **Table 1**. Recombinant *Amb a 1*, *Bet v 1*, *Phl p 2*, *Alt a 1*, and D*er p 1* were purchased from Indoor Biotechnologies Inc. (Charlottesville, VA).

**Table 1:** Allergen list.

Category	Common name	Scientific name	Abbr.
Grass pollen	Bermuda grass	*Cynodon dactylon*	Ber
	Bluegrass	*Poa pratensis*	Blu
	Johnson grass	*Sorghum halepense*	Jhn
	Orchard grass	*Dactylis glomerata*	Orc
	Ryegrass, Perennial	*Lolium perenne*	Rye
	Timothy grass	*Phleum pratense*	Tim
Weed pollen	Mugwort, Common	*Artemisia ambrosioides*	Mug
	Ragweed, Short	*Ambrosia artemisiifolia*	Rag
Tree pollen	Alder, European	*Alnus glutinosa*	Ald
	Ash, White	*Fraxinus americana*	Ash
	Birch, White	*Betula populifolia*	Bir
	Cedar, Mountain	*Juniperus ashei*	Ced
	Cottonwood, Eastern	*Populus deltoides*	Cot
	Elder, Box	*Acer negundo*	Eld
	Elm, American	*Ulmus americana*	Elm
	Mulberry, Red	*Morus rubra*	Mul
	Oak, Red	*Quercus rubra*	Rok
	Oak, White	*Quercus alba*	Wok
	Olive	*Olea europaea*	Olv
	Pecan	*Carya illinoensis*	Pec
	Sycamore, Western	*Platanus racemosa*	Syc
	Walnut, Black	*Juglans nigra*	Wal
Non-pollen	Alternaria	*Aspergillus niger*	Asp n
	Aspergillus	*Alternaria alternata*	Alt a
	Dustmite, American	*Dermatophagoides farinae*	Der f
	Dustmine, European	*Dermatophagoides pteronyssinus*	Der p
	Cockroach, American	*Periplaneta americana*	Acr
	Cockroach, German	*Blattella germanica*	Gcr
	Cat hair	*Felis catus*	Cat
	Dog epithelia	*Canis familiaris*	Dog
	Dust	*NA*	Dst

Abbr. – abbreviations.

To isolate pollen surface materials, raw un-defatted pollen was suspended in cyclohexane and vortexed. After separation by filtration through a glass filter, the cyclohexane was evaporated and the remaining material was resuspended in TBS-T (150 mM sodium chloride, 10 mM Tris base, pH 7.4, 1% Tween-20). Proteins were precipitated with 80% ice-cold acetone and the pellets were washed until white.

To isolate the pollen cytoplasm, washed pollen (from previous step) was hydrated with TBS and mechanically pulverized with sea sand (Fisher, Pittsburgh, PA). After separation by filtration through a glass filter, proteins were precipitated with 80% ice-cold acetone and pellets were washed until white.

Pellets were resuspended in suspension buffer (50% Protein Printing Buffer (ArrayIt, Sunnyvale, CA), 25% TBS-T, 10% glycerol). All protein solutions, including the commercial allergen extracts, were quantified with BCA Protein Assay Kit according to manufacturer's protocol (Pierce, Rockford, IL) and diluted to a concentration of 1 ug/uL in suspension buffer.

### Serum samples

Sera from de-identified individuals with high levels of total IgE (>300 kU/L) and pooled sera from 500 randomly selected individuals were purchased from Bioreclamation Inc. (East Meadow, NY). Additional sera from 76 de-identified patients were obtained from the University of Chicago *in vitro* allergy laboratory.

### SDS-PAGE and dot blots

Commercial allergen extracts and cytoplasmic and surface protein fractions were separated by single dimension SDS-PAGE using 5% stacking and 12.5% separating gels. Gels were stained with Coomassie blue according to standard protocols.

For the dot blots, 1 uL of pollen fractions were spotted onto nitrocellulose membranes at a concentration of 2 ug/uL. Dried membranes were washed, blocked with PBS-T containing 5% dry milk, and incubated with 20% pooled human sera overnight at 4°C followed by human anti-IgE secondary antibody conjugated with horseradish peroxidase (HRP) (Serotec, Raleigh, NC). Bound antibodies were detected using SuperSignal West Pico Chemiluminescent kit using manufacturers' protocol (Pierce, Rockford, IL). Resulting chemiluminescence was detected on Kodak BioMax XAR film (Fisher, Pittsburgh, PA). Washing with PBS-T was done between all steps.

### ImmunoCAP RAST

ImmunoCAP RAST data from allergic patients were obtained from the University of Chicago *in vitro* allergy laboratory. The allergens tested included D. pteronyssinus, D. farinae, German cockroach, Cat epithelium and dander, Dog epithelium, Aspergillus fumigatus, A. alternaria, Meadow fescue, Timothy, Elder, Maple leaf sycamore, Cottonwood, White ash, Cedar, White Oak, Lamb's quarters, Common pigweed, Rough marshelder, Sheep sorrel, Ribwort, and (giant) Ragweed.

### Allergen-specific IgE ELISA

Sera were screened by ELISA for specific IgE to commercial extracts and cytoplasmic protein fractions from six pollens (bermuda grass, Timothy grass, mugwort, ragweed, birch, and cottonwood). Maxisorb plates (NUNC, Rochester, NY) were coated with allergens at a concentration of 5 ug/mL in PBS and wells reserved for standards were coated with anti-human IgE (KPL, Gaithersburg, MD) at a concentration of 2 ug/mL in PBS. Plates were incubated overnight at 4°C. Plates were then washed three times with PBS-0.05% Tween 20, blocked for 120 minutes with 10% fetal bovine serum (FBS) in PBS at room temperature, and then washed three times with PBS-0.05% Tween 20. A standard serial dilution (20, 10, 5, 2.5, 1.25, 0.625, 0.3125 and 0 ng/mL) of purified human IgE (Fitzgerald, Concord, MA) was added to wells reserved for standards, serum samples were added to the rest of the plate at a 1∶4 dilution in 10% FBS in PBS and incubated overnight at 4°C. Plates were then washed three times with PBS-0.05% Tween 20, followed by incubation with mouse anti-human IgE conjugated to HRP (Serotec, Raleigh, NC) at a concentration of 1 ug/mL for 60 minutes a room temperature. After washing the plates four times with PBS-0.05% Tween 20, SureBlue TMB 1-Component Microwell Peroxidase Substrate (KPL, Gaithersburg, MD) was added according to manufacturers' protocol and the reaction was stopped with TMB stop solution (KPL, Gaithersburg, MD) after sufficient color development. Adsorbance was read at 450 nm on a plate reader and the amount of antibody binding was extrapolated from the standard calibration curves. Positive signal cutoff for ELISA was signals >0.35 kU/L and positive signal cutoff for microarrays was signals greater than the allergen specific threshold calculated from mock arrays.

### Allergen microarray fabrication

Solubilized protein fractions were printed in microarray format with 12 microarrays per standard SuperEpoxi glass slide (using ArrayIt Stealth Printing technology). Microarrays contained a total of 80 allergens printed in triplicate: pollen surface and cytoplasmic protein fractions and commercial extracts from the pollen of six grasses, two weeds, and 13 trees; commercial extracts of cat hair, dog epithelia, dust, two mites, two cockroaches, and two molds; and five recombinant major allergens. Negative controls (human serum albumin (HSA) and buffer) and standard calibration curves of purified IgE (Fitzgerald), IgG, and IgA (Bethyl) at 200, 100, 50, 25, 12.5, 6.25, 3.125, and 1.5625 pg/spot were printed in six replicates. Microarrays were stored at room temperature in vacuum-sealed boxes until use.

### Allergen microarray immunoassay

Slides containing allergen arrays were washed with PBS-T, rinsed with ddH_2_O, blocked for 120 minutes at room temperature with BlockIt buffer (ArrayIt, Sunnyvale, CA), rinsed with ddH_2_O, and washed with PBS-T. Gaskets (Grace BioLabs, Bend, OR) were attached to the slides to create a barrier between arrays, and a total of 50 uL of serum diluted to 25% in PBS-T containing 1% HSA (HSA, Sigma, St. Louis, MO) was applied to the individual reaction wells, which were then sealed to prevent evaporation. After incubation with sera overnight at 4°C, the slides were washed with PBS-T and rinsed with ddH_2_O. To detect bound IgE antibodies, the slides were incubated for 120 minutes at 37°C with anti-human IgE labeled with Alexa Fluor 647 (Molecular Probes, Carlsbad, CA) diluted 1∶100. Subsequently, slides were washed with PBS-T followed by PBS, rinsed with ddH_2_O, spin-dried, and stored in the dark until scanning.

### Scanning, quality control, and data processing

Slides were scanned using an Axon GenePix 4000B scanner (Sunnyvale, CA) and images were analyzed using GenePix Pro 6.0 software to obtain median foreground intensity values for both red (635 nm) and green (532 nm) channels. Irregularly shaped, smeared, and missing spots were flagged. An automated data processing program was developed in R version 2.5.0 (available at http://www.r-project.org) to 1) discard flagged spots, 2) log 2 transform data, 3) correct for autofluorescence of spots, and 4) determine allergen-specific thresholds. The IgE standard calibration curves were used to make sure that the secondary antibodies were property working but were not used for normalization or extrapolation of the amount of IgE binding to allergen spots because this introduced unacceptable error into our calculations.

**Figure 1:**
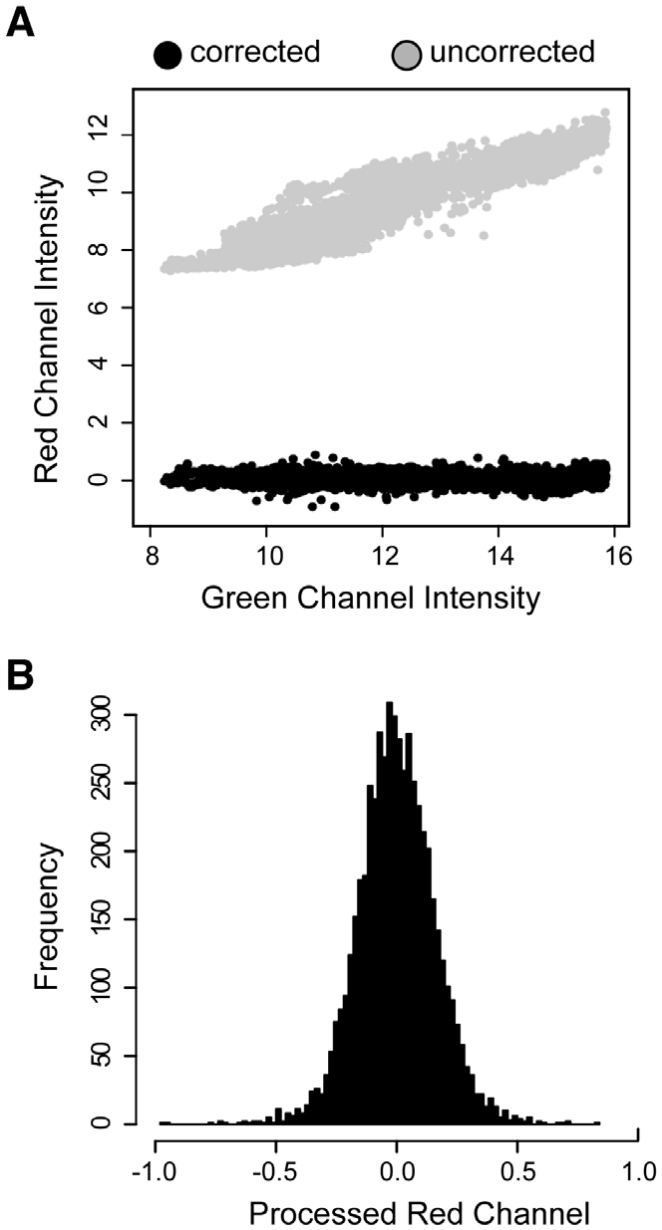
Autofluorescence correction of fluorescence intensity values. **A**) Scatter plot of green vs. red channel data of uncorrected (grey circles) and autofluorescence-corrected (black circles) fluorescence intensity values. **B**) Histogram of autofluorescence-corrected fluorescence intensity values on 36 mock arrays probed with secondary antibody only (no sera).

Autofluorescence in both the red and green channels, which varied from spot-to-spot and allergen-to-allergen, was corrected using the assumption that for each spot, the red channel intensity *(R)* is the sum of autofluorescence *(R_AF_)* and the fluorescence of the bound secondary antibody *(R_IgE_)*. The green channel intensity *(G)* was not affected by the binding of the secondary antibody (data not shown), and was entirely the result of autofluorescence *(G_AF_)*. We observed a linear relationship between red and green channel fluorescence on slides incubated with buffer alone (**Fig. 1a**), thus *R_AF_ = mG_AF_+b*, where *b* is an experimentally determined constant. We estimated *R_AF_* from *G_AF_* on arrays containing sera by applying linear models for each allergen separately, and this value was then subtracted from *R* to obtain *R_IgE_*. When mock slides were processed in this manner, the distribution of signal intensities was tightly centered at 0 (**Fig. 1b**).

To avoid an excess of false positive signals, allergen-specific thresholds were determined as the value at the 99^th^ percentile from the distribution of corrected fluorescence intensity values on mock arrays for each allergen. These values were then subtracted from corresponding values on sample arrays, resulting in a 1% false positive rate. Finally, allergens with >50% missing data were discarded and results were recorded in microarray units corresponding to the Tukey mean of corrected fluorescence intensity of replicates.

### Statistical Analyses

Coefficient of variances (CV) were calculated as the standard deviation divided by the mean of replicates. To determine intra-slide reproducibility, individual sera were tested on six different arrays on the same slide. Inter-slide variation was measured by testing these sera on three different slides on the same day. Inter-assay reproducibility was determined by testing the same sera on three different days. Median CV of six individuals tested for 80 allergens each where the mean of the replicates was >0.5 were reported to avoid skews to the data when there were small differences among low values.

Analysis of Variance (ANOVA) and correlation analyses between pair-wise replicates were carried out using standard functions in R. To compare the relative reactivity to different pollen fractions, we used t-tests for matched pairs, testing the null hypothesis of no difference between the means. Two tailed p-values are reported.

Hierarchical clustering analysis of allergen-specific IgE was performed in R version 2.5.0 using package *pvclust* [34] with the following parameters: agglomerative method - average, distance measure - correlation, number of bootstrap replicates - 1000. *pvclust* provides two types of p-values: AU (Approximately Unbiased) p-value, computed by multiscale bootstrap resampling and BP (Bootstrap Probability) value, computed by normal bootstrap resampling.

## Results

### Allergenicity of different types of pollen fractions

**Figure 2:**
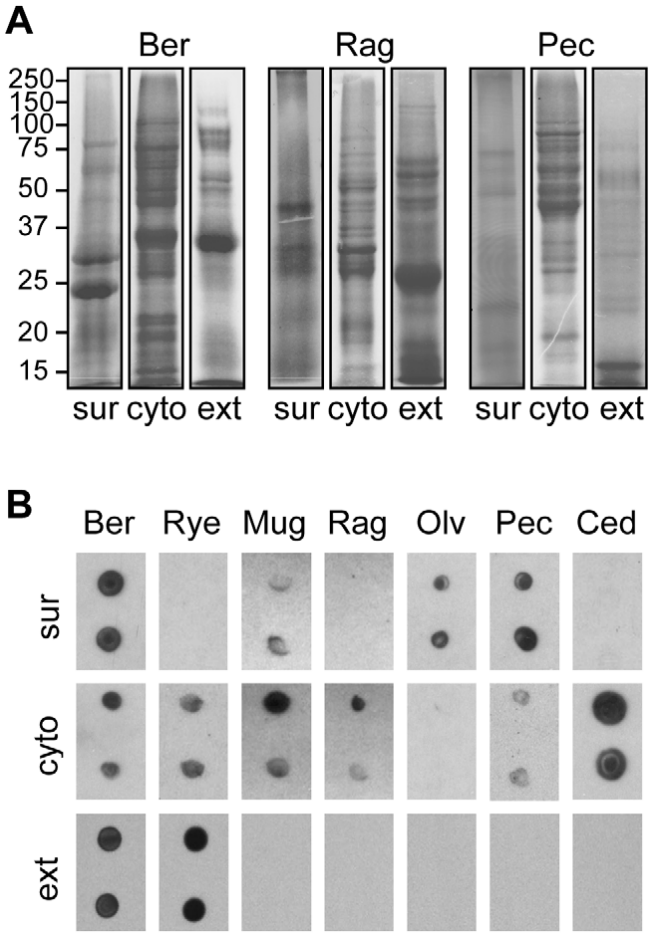
Comparison of surface fractions, cytoplasmic fractions, and commercial allergen extracts. **A**) SDS-PAGE gels stained with Coomassie blue. **B**) Nitrocellulose dot blots probed with sera pooled from 500 individuals and HRP-conjugated anti-IgE.

To fractionate pollen into its different components, we isolated pollen surface materials by extraction into cyclohexane [28] and obtained cytoplasmic fractions by the pulverization of pollen, followed by acetone precipitation of proteins. Precipitated proteins were solubilized and, in addition to commercial allergen extracts, visualized on Coomassie-stained SDS-PAGE. For three different species examined (Bermuda grass, ragweed and pecan) the extracts prepared from the pollen surface and cytoplasm contained distinct bands, with numerous proteins that were absent from commercially purchased pollen extracts (**Fig. 2a**). The differences in the content of the commercial and cytoplasmic extracts, which were both derived from washed pollen, are somewhat surprising, and may be attributable to different extraction methods, protein solubilization, or protein stability.

Next, we tested whether the pollen fractions contain candidate allergens by spotting equivalent extract quantities (normalized to total protein) onto nitrocellulose membranes and blotting with human sera pooled from 500 American individuals of diverse age and ethnicity. Specific IgE binding, as detected by an anti-IgE monoclonal antibody, was observed with each of the seven pollen species tested, including strong signals to four pollen surface fractions (Bermuda grass, mugwort, olive, and pecan), six cytoplasmic fractions (Bermuda grass, ryegrass, mugwort, ragweed, pecan, and cedar), and two commercial extracts (Bermuda grass and ryegrass) (**Fig. 2b**). Further characterization of allergens contained within IgE-reactive surface fractions is described elsewhere (manuscript in preparation).

### Development and validation of the allergen microarray

**Figure 3:**
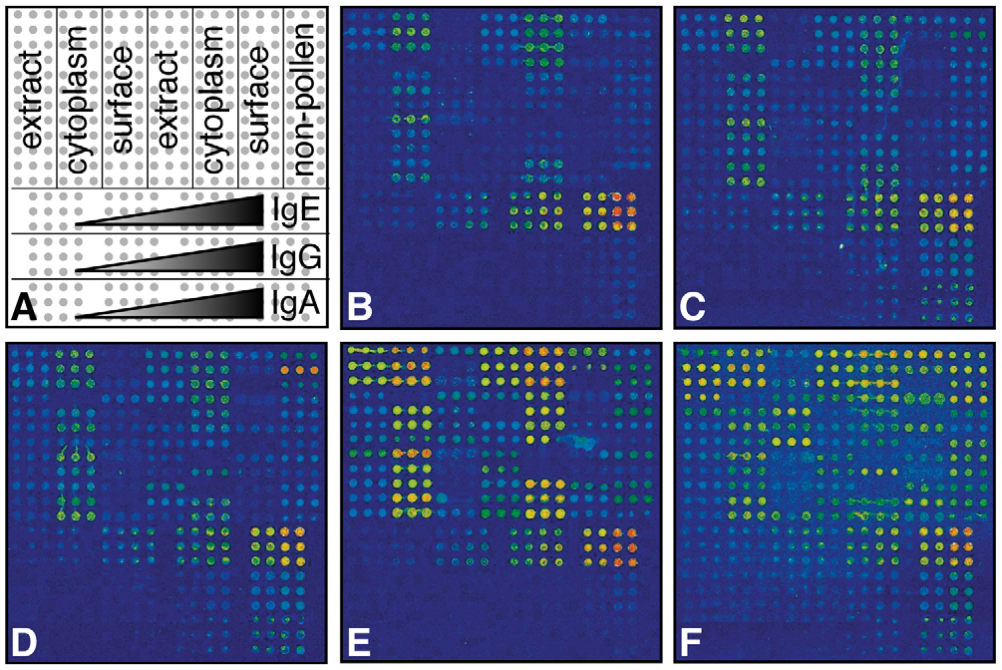
IgE profiling using protein microarrays. **A**) General layout of the allergen microarray, **B**) scanned image of an array probed only with secondary antibody to show autofluorescence, **C–F**) and scanned images of arrays probed with sera from four individuals showing different allergen sensitization profiles. Images were pseudocolored with a color spectrum adjusted so that blue indicates low signal and red indicates high signal.

**Figure 4:**
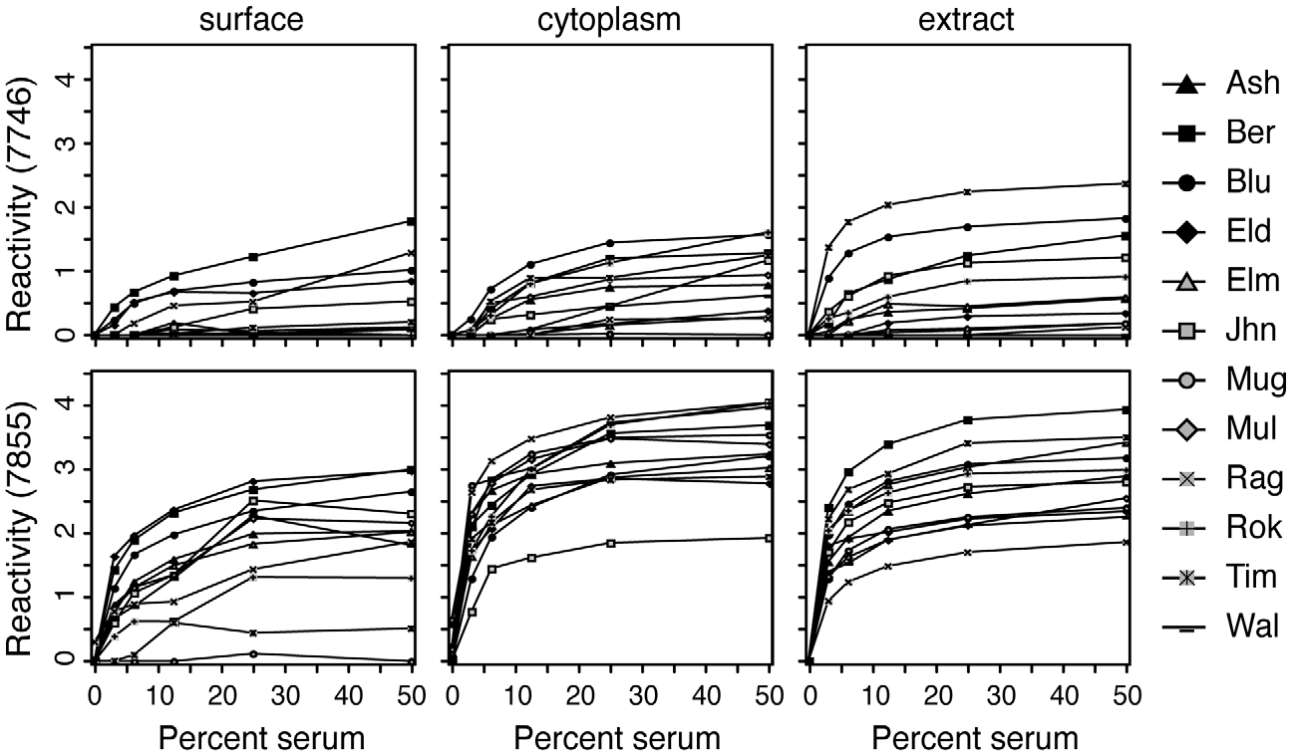
Serum dilution vs. fluorescence intensity values. Two serum samples, one with moderate levels of allergen-specific IgE (7746) and one with high levels of allergen-specific IgE (7855) were tested at five different dilutions; 50%, 25%, 12.5%, 6.25%, and 3.125%. Specific IgE in microarray units to the three fractions (surface, cytoplasm, and commercial extract) of 12 pollen allergens is shown.

To make a high-throughput assay testing allergen-specific IgE in sera, microarrays were printed containing protein fractions described above. We incubated the allergen microarrays with patient sera, and demonstrated that our method can successfully distinguish among serum samples. **Figure 3** shows that some individuals have specific IgE to almost all allergens (**e,f**), while others to only a few (**d**) or none at all (**c**). Diluting two serum samples over a range of 50% to 3%, showed a predictable and consistent decrease in signal intensity, with similar trends across different allergen spots (**Fig. 4**).

**Figure 5:**
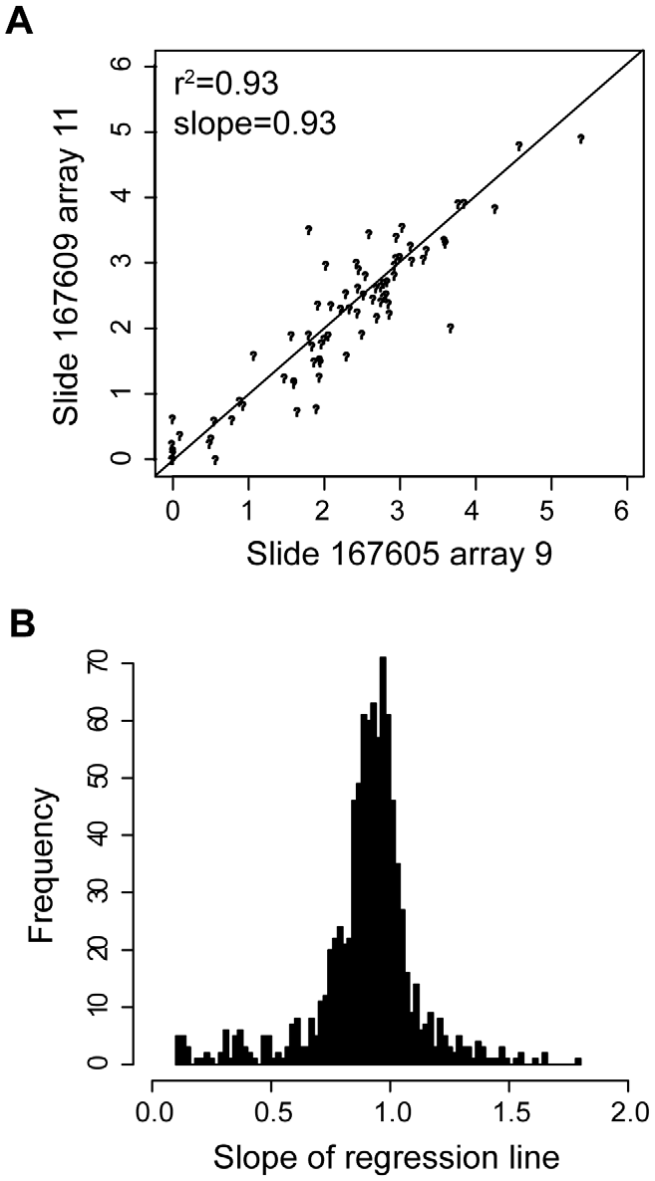
Reproducibility of the allergy microarray. Six individuals were serially tested 18 times for 80 allergens on the allergen microarray. **A**) Representative correlation plot of corrected fluorescence intensity values (circles) of one serum sample binding to 80 allergens on two replicate arrays. **B**) Histogram of slopes of regression curves calculated for 918 pair-wise comparisons of replicates.

We assessed the reproducibility of the microarray, by performing replicate tests using sera from six individuals with a wide range of allergen-specific IgE levels. Analysis of variance (ANOVA) showed that the identity of the allergen and source of the serum were responsible for most of the observed variation in the data (16.71% and 50.09%, respectively), and that array-to-array and slide-to-slide effects accounted for only 0.12% and 0.85%, of the variation respectively (**Table 2**). Regression analysis indicated that pair-wise replicates were highly correlated (median R^2^ = 0.93, **Fig. 5a**) and across all replicate pairs, the median of the slope of the regression (beta) was 0.93 (**Fig. 5b**). Interestingly, 80.33% of outlying values for beta, represented individuals who had overall very low allergen-specific IgE indicating that concordance was even higher in samples where true signals overpower noise. Median coefficient of variances (CV) measured 0.09 to 0.15 for comparisons of six arrays on the same slide, 0.11–0.20 for comparisons between slides, and 0.14–0.25 for comparisons between assays performed on three consecutive days (**Table 3**), all within the variance reported previously for recombinant allergen arrays [4], [16]. Measurements of CV were not statistically different for our cytoplasmic pollen extracts, commercial extracts, or recombinant major allergens. Pollen surface fractions, however, showed significantly more variability (p<0.05), perhaps reflecting protein aggregation or instability. Nonetheless, the reproducibility we observed across arrays, coupled with the strong correlation between signals and serum concentration, indicate that the allergen microarray can measure allergen-specific IgE over a wide range of concentrations.

**Table 2:** ANOVA of replicates.

	SS	df	% SS_T_
Array	51.191	11	0.12%
Slide	363	8	0.85%
Serum	21378	5	50.09%
Allergen	7132	78	16.71%
Error	13758	25155	32.23%

Analysis of variance statistics (ANOVA) of fluorescence intensity values; SS, sum of squares; df, degrees of freedom; SS^T^, percent of the total sum of squares.

**Table 3:** CV of replicates.

	Intra-slide	Inter-slide	Inter-assay
Surface	0.15	0.20	0.25
Cytoplasm	0.11	0.11	0.14
Pollen extract	0.09	0.11	0.17
Non-pollen extract	0.10	0.15	0.19
Recombinant	0.14	0.16	0.23

Median coefficient of variation (CV) of fluorescence intensity reported for pollen surface fractions (n = 21), pollen cytoplasmic fractions (n = 21), pollen commercial extracts (n = 21), non-pollen commercial extracts (n = 9), and recombinant allergens (n = 5). Only values where the mean of the replicates was >0.5 were analyzed.

**Figure 6:**
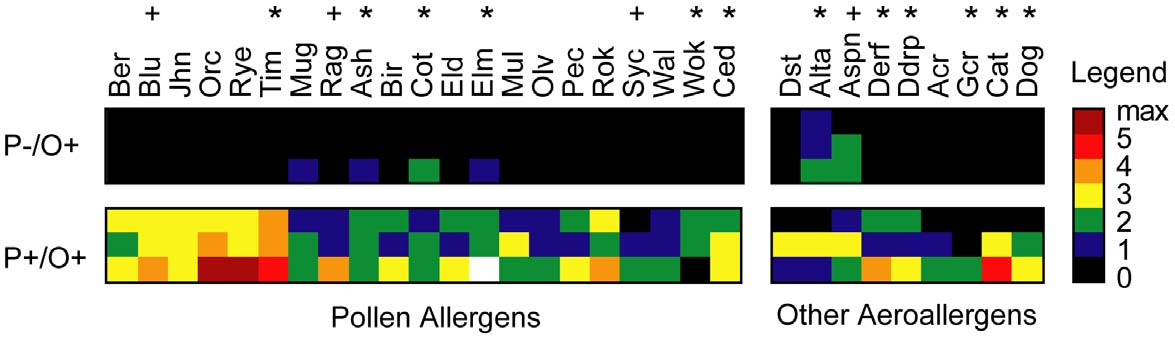
Allergen microarray testing vs clinical diagnosis of patients. Heatmap depiction of microarray results of six patients who were diagnosed as either not having pollen allergy, but having some other aeroallergy (P−/O+) or as having both pollen and other aeroallergy (P+/O+). Diagnoses were made at the *in vitro* allergy lab by ImmunoCAP RAST on 12 of the same (*) allergen species as on the array, four similar (+) allergen species as on the array, and five other weeds not present of the array. All other allergens on the allergen array were not tested by ImmunoCAP RAST.

To evaluate the clinical validity of our method, we tested sera from six patients who were diagnosed at the University of Chicago *in vitro* allergy lab as having either i) no pollen allergy, but some other aeroallergy or ii) both pollen and other aeroallergy. These diagnoses were based on ImmunoCAP RAST (see Methods). We observed that the allergen microarray results reflect the clinical diagnoses of patients, detecting pollen allergen-specific IgE only in patients who were diagnosed as pollen allergic (**Fig. 6**).

To further test the clinical validity of our method, we compared results obtained by the allergy microarray with those obtained by ELISA, an assay commonly used for clinical diagnosis. We measured specific IgE in 40 human sera, observing binding to cytoplasmic protein fractions and commercial extracts from six allergens: grasses (Bermuda grass and Timothy grass), weeds (mugwort and ragweed), and trees (birch and cottonwood) (see Methods). The allergen microarray detected a positive signal for 81% (26/32) and 97% (29/30) of the signals detected by ELISA for the cytoplasmic fraction and commercial extracts, respectively. However, for about 50% of cases, IgE was detected with the allergen microarray, but not with ELISA (**Table 4**). While it is possible that these signals are false positives, our conservative approach for setting thresholds (which controls the false positive rate at 1%) supports the alternate possibility that the microarray is more sensitive than ELISA. Further work using well-characterized patients is needed to fully compare these assays.

**Table 4:** Concordance of IgE detection by allergen microarray and ELISA.

	Cytoplasmic fraction			Commercial extract		
	ELISA+	ELISA−	Total	ELISA+	ELISA−	Total
ARRAY+	26 (0.11)	116 (0.48)	142 (0.59)	29 (0.12)	135 (0.56)	164 (0.68)
ARRAY−	6 (0.03)	92 (0.38)	98 (0.41)	1 (0.01)	75 (0.21)	76 (0.32)
Total	32 (0.14)	208 (0.86)	240 (1.0)	30 (0.13)	210 (0.87)	240 (1.0)

Specific IgE was measured for six allergens in 40 human sera using ELISA, an assay commonly used for clinical diagnosis. Cytoplasmic fractions and commercial extracts of each allergen were assayed. We scored each serum as positive (+) or negative (−) for specific IgE to each allergen by ELISA and allergen microarray using empirically defined detection thresholds (Methods). The results are cross-tabulated here, showing the counts of assays positive by one technique, positive by both techniques, and negative by both techniques (proportion of total assays in parentheses).

### Hierarchical clustering of allergen-specific IgE levels

We used the allergen microarray to investigate allergen-specific IgE in two populations that are likely to be seen in an allergy clinic: 100 individuals with high levels of total IgE (>300 kU/L) and 76 allergic individuals from the *in vitro* allergy lab at the University of Chicago.

**Figure 7:**
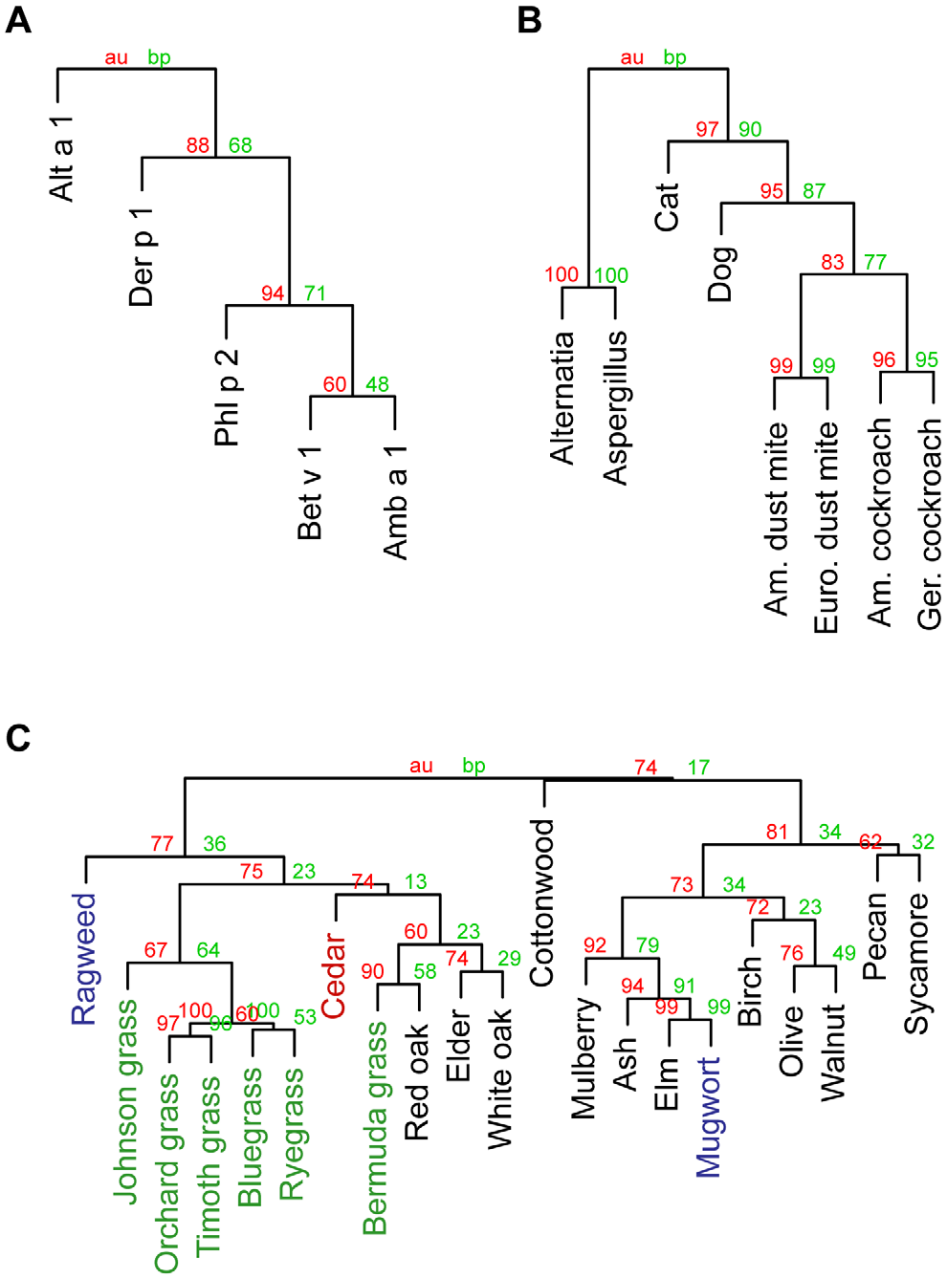
Clustering of allergen-specific IgE levels. Hierarchical clustering of allergen-specific IgE to **A**) recombinant, **B**) non-pollen, and **C**) pollen allergens. Within the pollens, grasses are indicated in green, trees in black, weeds in blue, and cedar (a gymnosperm) in red.

To examine possible patterns in allergen-specific IgE, we performed hierarchical clustering on allergen microarray-generated data. Analysis of specific IgE to five recombinant allergens, which included a mite (*Der p 1*), a mold (*Alt a 1*), a grass pollen (*Phl p 2*), a weed pollen (*Amb a 1*), and a tree pollen (*Bet v 1*), revealed a striking clustering according to the phylogeny of the allergen source. The three pollens clustered together, but differentiated between monocots (*Phl p 2*) and dicots (*Amb a 1* and *Bet v 1*) (**Fig. 7a**). When this analysis was limited to only non-pollen allergen extracts, including two molds and six animals including two mites, two cockroaches, and two mammals (cat and dog), a similar pattern was observed. Remarkably, not only did the pairs of most closely related species cluster together, but the dendrogram reconstructed the phylogeny of these species, with the molds clustering away from the animals, and within the animals, the arthropods (mites and cockroaches) clustering away from the mammals (**Fig. 7b**). Although clustering was less pronounced when the analysis was limited to only pollen allergens, five of the six grasses, which are monocots clustered together away from trees and weeds, which are dicots (**Fig. 7c**). Although these results could be explained by cross-reactivity [35], [36], the fact that the allergen microarray-generated data followed these patterns is quite surprising. Previous studies examining cross-reactivity among allergens have studied similarity of sequence and protein structure among individual proteins, while we test for specific IgE to a mixture of potentially allergenic proteins. Additionally, studies have indicated that phylogenetically distantly related species such as mite and shrimp can be cross-reactive due similar allergenic proteins [37]. Although it is possible that there is one major allergen that is driving the clustering of allergen-specific IgE data, it is more likely that a combination of several related proteins contributes to the clustering that we observed.

### Comparison of specific IgE to surface, cytoplasmic and commercial extracts

**Figure 8:**
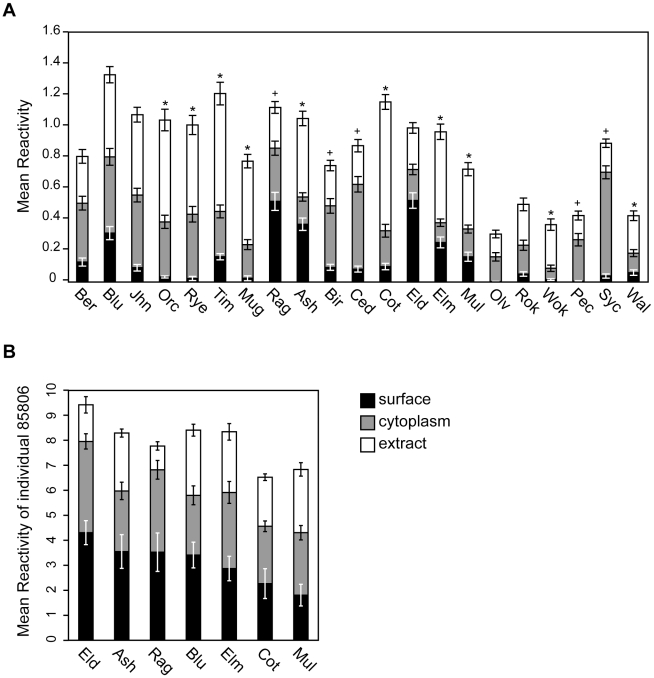
Comparison of specific IgE to surface, cytoplasmic and commercial extracts. Bar graphs comparing levels of specific IgE in microarray units to surface fractions (black), cytoplasmic fractions (grey), and commercial extracts (white) of different pollen allergens. **A**) Bars represent the mean of 176 individuals with standard error bars. Cases where specific IgE to commercial extracts was significantly greater than to cytoplasmic extracts are denoted by a black asterisk (*) and cases where specific IgE to cytoplasmic fractions was significantly greater than to commercial extracts is denoted by a plus sign (+). **B**) Bars represent the mean of 18 replicates (of individual 85806) with standard error bars.

Comparison of the relative amounts of specific IgE among individuals to the different pollen extracts (surface, cytoplasm, and commercial extract), revealed that, as a class, the amount of specific IgE to cytoplasmic proteins was not significantly different from the amount of specific IgE to commercial pollen extracts (p>0.10). However, many individual allergens showed differences, including those tested in the dot blot experiments (**Fig. 8a**). Among these, individuals had significantly higher amounts of specific IgE to the cytoplasmic fraction of ragweed (p = 1.59×10^−3^), pecan (p = 4.71×10^−4^), and cedar (p = 1.10×10^−11^) as compared to the commercial extracts. In contrast, no significant differences were noted for Bermuda grass or olive, while for ryegrass, the amount of specific IgE to the commercial extract was significantly higher than to our cytoplasmic fraction (p = 2.11×10^−8^). For other allergens that were not tested on the dot blots, the amount of specific IgE to commercial extracts tended to be higher than to cytoplasmic fractions, perhaps reflecting greater purity or a higher proportion of allergenic proteins to non-allergenic proteins in these particular batches of commercial extracts (**Fig. 8a**).

While the amount of specific IgE to pollen surface was not as high as to other fractions, a substantial number of individuals (25%) had high amounts (>1.0 microarray units) to at least one surface extract. The most allergenic surface allergens were elder, ragweed, ash, bluegrass, and elm (**Fig. 8a**). In fact, some serum samples showed extensive sensitization to surface fractions, with cases of higher amounts of specific IgE to surface fractions as compared to commercial extracts (**Fig. 8b**). These data are consistent with observations made on the gels and dot blots, indicating that the pollen surface, which has been largely overlooked, contains allergens. Lack of detectable specific IgE to certain surface fractions could be due to the serum population used or to less efficient extraction of proteins from these species. Indeed, due to the highly lipophilic nature of pollen surface proteins, effective isolation remains challenging.

### Comparison of specific IgE to recombinant allergens and total protein extracts

**Figure 9:**
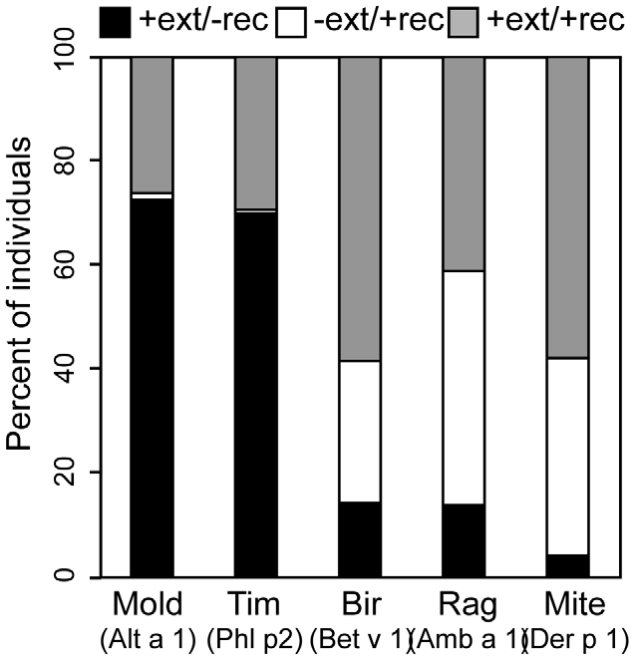
Comparison of specific IgE to recombinant allergens and total protein extracts. Bar graph comparing reactivity to commercial extract vs. the corresponding recombinant major allergens in microarray units. Bars represent the percent of individuals (mold: n = 153, Tim: n = 143, Bir: n = 106, Rag: n = 124, Derp: n = 171) who show positive reactivity to commercial extract but no reactivity to recombinant major allergen (black), positive reactivity to recombinant major allergen but no reactivity to commercial extract (white), and positive reactivity to both (grey).

To directly test the utility of screening recombinant allergens, rather than extracts containing a mixture of proteins, we included five recombinant major allergens on the allergen array; *Bet v 1* from birch, *Amb a 1* from ragweed, *Phl p 2* from Timothy grass, *Der p 1* from mite, and *Alt a 1* from mold. While the recombinant major allergen from mite, ragweed, and birch captured over 80% of sensitized individuals, for all species, there were several individuals who had specific IgE to commercial extracts, but not to the corresponding recombinant allergen. The least informative recombinant allergens were from mold (*Alta a 1*) and Timothy grass (*Phl p 2*) for which 73% and 70% of allergic individuals had specific IgE to commercial extracts but not the recombinant major allergen, respectively (**Fig. 9**). These results imply the presence of another allergen in these species to which the majority of people have specific IgE. Alternatively, post-translational modifications present in plant cell extracts may be missing in the recombinant proteins, especially if they were expressed in a non-eukaryotic system. Conversely, 27–45% of individuals had specific IgE to the major recombinant allergen from birch (*Bet v 1*), ragweed (*Amb a 1*), and mite (*Der p 1*), but not to the total protein extracts. These results suggest that the relative concentration of the major allergen is substantially lower in the total protein extract than the recombinant allergen.

## Discussion

Here, we developed a protein microarray containing 80 different allergen fractions from 31 different species of allergen sources, and have demonstrated that it can reproducibly measure allergen-specific IgE in small amounts of sera. With the continuing advancement of protein microarray technology, this microarray could be expanded to hundreds or even thousands of allergens, providing an efficient and economically feasible assay to measure allergen-specific IgE in sera.

Previous studies explored this possibility by focusing on a few recombinant allergens and have suggested that microarrays containing recombinant allergens offer advantages over those containing native protein extracts [4], [7], [9]–[11], [38]. They argue that 1) easier standardization of recombinant allergens results in better reproducibility across assays and 2) that recombinant allergens offer very specific diagnosis, identifying disease-eliciting molecules that can be used for more effective immunotherapy. However, we showed that our microarray is highly reproducible whether allergens are recombinant or present in native extracts. Additionally, limiting analysis to recombinant major allergens potentially restricts diagnosis to just over half of the affected population [39] and due to the lack of post-translational modifications, some recombinant allergens may not display the same immunological reactivity as their native counterparts [40]. Indeed, our results indicate that the recombinant major allergens from Timothy grass (*Phl p 2*) and mold (*Alt a 1*) are not sufficient to diagnose individuals for allergen sensitization. We also successfully showed that pollen surface fractions, which have largely been overlooked in the past, contain allergens. It is thus important to pursue the identification, cloning, and characterization of these pollen surface allergens, and this is the subject of another manuscript (in preparation). Nonetheless, the utility of recombinant allergens remains clear, especially with regard to molecule-specific diagnosis. Microarrays containing combinations of extracts and recombinant allergens will likely be the most beneficial diagnostic approach, combining comprehensive testing and protein-specific analysis, as suggested by Fall and colleagues [8].

Further refinement of the allergen microarray will likely improve its utility for clinical and research applications. For example, we observed significant autofluorescence that varied from spot-to-spot and allergen-to-allergen, possibly caused by differential spotting of fluorescent proteins, aggregated proteins, or non-proteinaceous fluorescent contaminants in the extracts. While a computational approach effectively corrected for autofluorescent signals, some variation was undoubtedly introduced by this phenomenon. Improved purification, solubilization, and quantification of proteins spotted on the microarray would likely make this a more reliable assay. The use of a two-color antibody labeling system similar to that described by Kattah and colleagues [41], could also reduce variability. Finally, although we have shown that our method reflects clinical diagnosis of patients (as determined by ImmunoCAP RAST) and displayed high concordance with ELISA in detecting the presence of allergen-specific IgE, it is still important to establish the extent of the allergen microarray's reliability and clinical relevance by validating it using large populations of well-characterized allergy patients. Ultimately, the use of the allergen microarray in conjunction with assessment of symptoms by a doctor, could greatly improve the accuracy and efficiency of allergy diagnostics.

In addition to its application in the clinic, the allergen microarray can be used as a research tool to quickly collect phenotype data for genetic mapping of susceptibility genes. Genetic studies have implicated several genes in allergy disposition, but most have not been consistently replicated across populations due to the complex nature of the disease [42]–[44]. Measuring allergen-specific IgE with the allergen microarray may facilitate quantitative trait analysis of allergy predisposition in populations, particularly if the number of genetic and environmental factors influencing this intermediate phenotype is smaller than the number of factors affecting the complex disease [45], [46]. Indeed, asthma linkage and association studies have benefited from a similar approach [47].
